# Supplementary figures and images for: Comparative Transcriptome Analysis of Two Sugarcane Cultivars in Response to Paclobutrazol Treatment
Source: Plants (Basel). 2022 Sep 16;11(18):2417. doi: 10.3390/plants11182417 (PMC9502373; doi:10.3390/plants11182417)

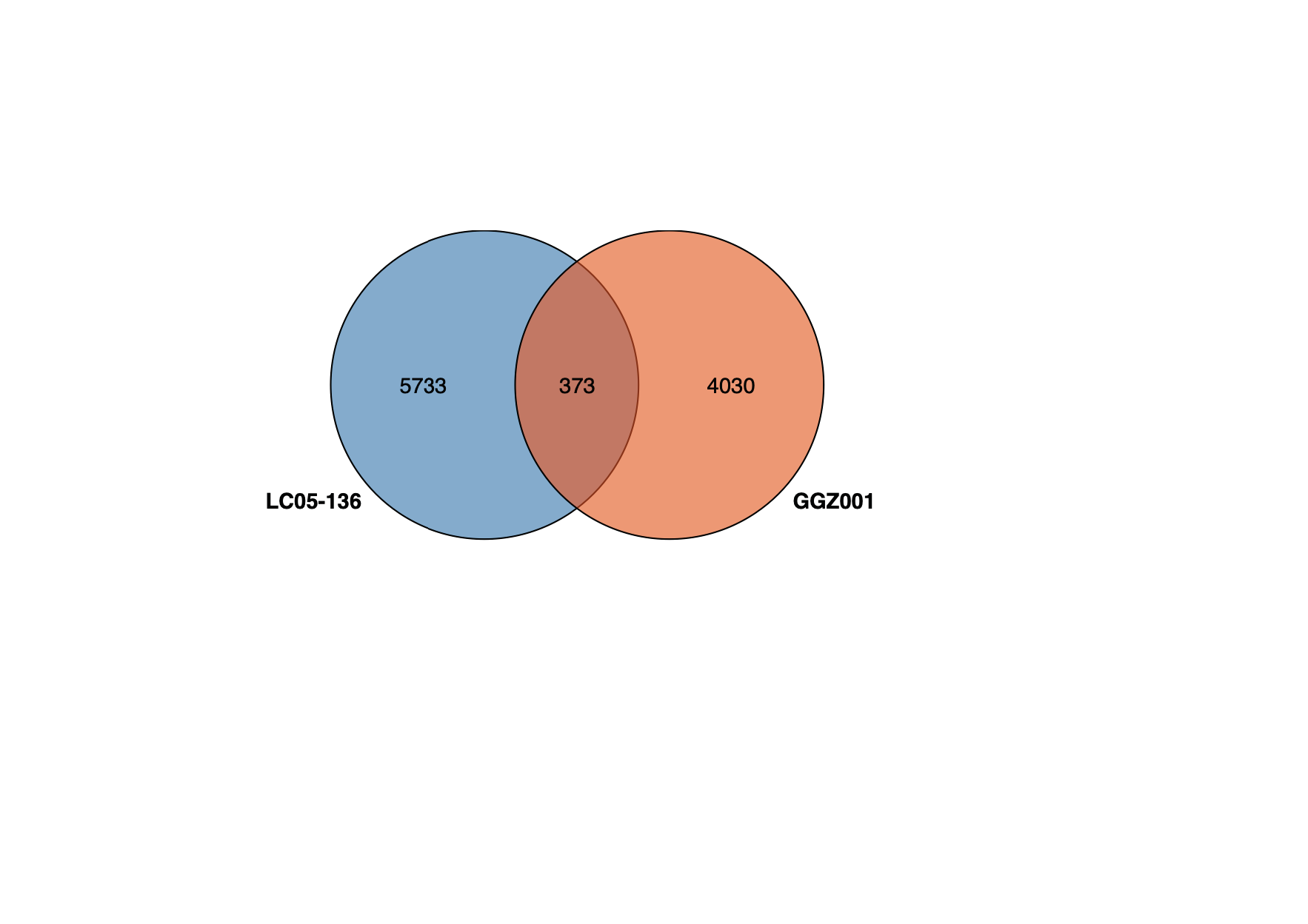

Supplement: Supplementary file 1 [file plants-11-02417-s001.zip › Figure S1.png]

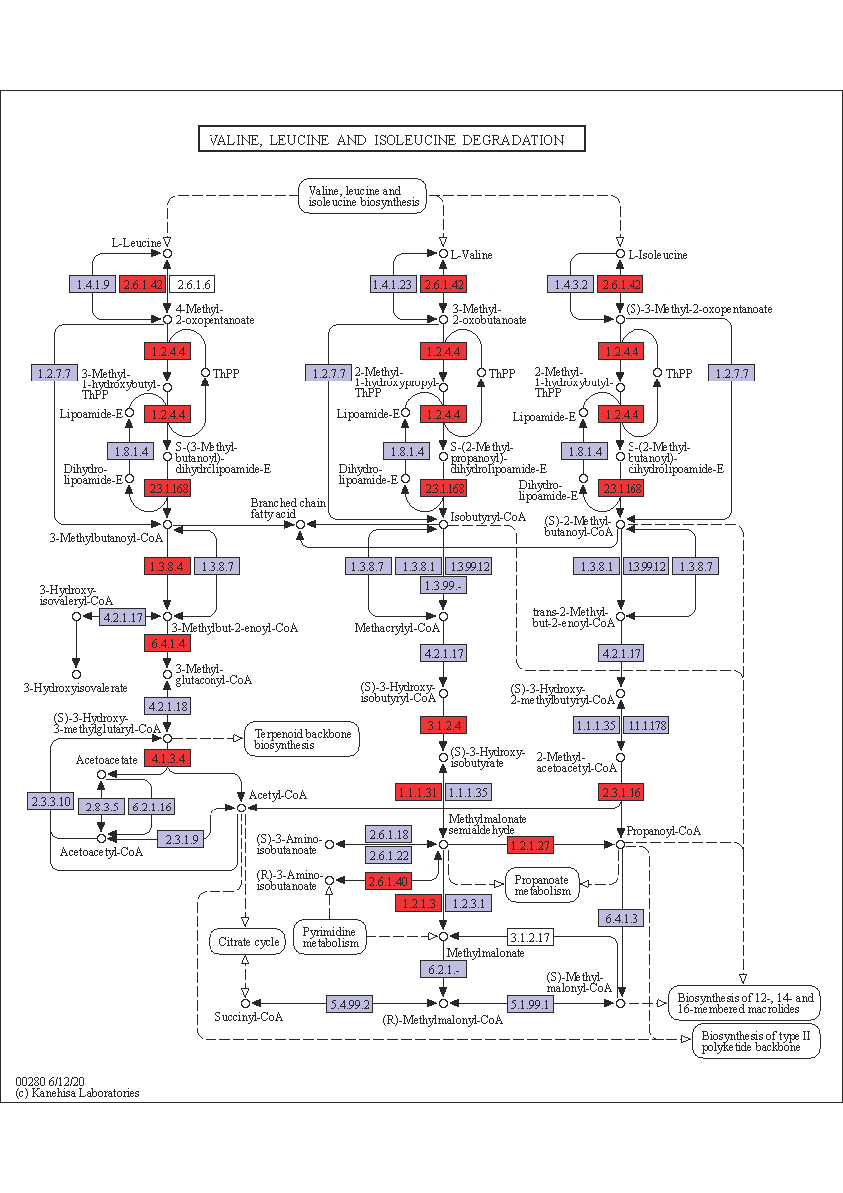

Supplement: Supplementary file 1 [file plants-11-02417-s001.zip › Figure S2.png]
